# Long COVID Oral Cavity Symptoms Based on Selected Clinical Cases

**DOI:** 10.1055/s-0041-1739445

**Published:** 2021-12-17

**Authors:** Barbara Rafałowicz, Leopold Wagner, Juliusz Rafałowicz

**Affiliations:** 1 Department of Dental Propaedeutics and Prophylaxis, Medical University of Warsaw, Warsaw, Poland; 2 Stomatologia Rafałowicz, Warsaw, Poland

**Keywords:** changes of mucosa, tongue, palate, cheilitis, long COVID

## Abstract

Severe acute respiratory syndrome coronavirus-2 (SARS-CoV-2) is characterized by variable clinical features, different durations, and several previously unheard-of late complications. Knowledge about this infection is constantly evolving.

The aim of the study is to present selected cases of the most common symptoms of long COVID in the oral cavity.

Among the 1,256 studied patients, 32% of them had discoloration, ulceration, and hemorrhagic changes on the oral mucosa, 29.69% had mycosis located on the tongue, 25.79% of patients had aphthous-like lesions on the hard palate, and in 12.5% atrophic cheilitis was observed. During the anamnesis, approximately 60% of patients reported salivary secretory disorders in the initial period of infection, which is 6.68% prolonged up to 4 months after systemic symptoms disappeared. In an extreme case, an aphthous-like lesion was located on the hard palate, which persisted for 6 months. Approximately 36% of patients did not agree to the proposed treatment. As a result, they only received recommendations on the use of oral hygiene products and received weekly check-ups. In this group of patients, most pathological changes spontaneously cleared after 3 weeks. The elderly with coexisting diseases, persons with a more severe SARS-CoV-2, and hospitalized patients had more extensive and severe lesions in the oral cavity that persisted for a long time after infection.

In patients after the SARS-CoV-2 infection and suspected of this infection, a detailed intraoral examination should be performed, and the patient must be obligatorily monitored for a minimum period of 6 months. Depending on the patient's clinical condition, changes in the oral cavity require observation, basic or specialist treatment. In the case of changes in the cavity without pain symptoms, observation should be made for approximately 4 weeks and wait for the spontaneous regression of the changes. However, when pain occurs, a good solution is to use laser biostimulation. In the case of complex pathological changes occurring in the oral cavity, the patient should be directed for specialist treatment.

## Introduction

The severe acute respiratory syndrome coronavirus-2 (SARS-CoV-2) pandemic has implemented a new reality affecting patients and health care professionals. The dentists who work in the immediate epidemic danger zone are also at risk.

Until now, many articles on diagnostics, virus spread, and the role of saliva in its transmission have been published. 1 2 3

SARS-CoV-2 is most often accompanied by fever, fatigue, malaise, conjunctivitis, dry cough, myalgia, arthralgia, headaches, throat pain, dysgeusia and smell, sickness, diarrhea, dyspnea, pneumonia, and organ dysfunction. 1 2 There are also skin symptoms, e.g., maculopapular and erythematous rashes, widespread urticaria, chickenpox-like vesicles, pseudo-chilblains, blotch, petechiae, distal ischemia, necrosis, livedo racemosa, and Stevens–Johnson syndrome. 4 In the case of the oral cavity, the most common symptoms are pain, dryness, erythema, various changes of mucosa and the lips, white plaque, mycosis, various changes of the tongue, swelling, herpetic lesions, pemphigus, lichen planus, and Sjögren's syndrome. 5 6 7

After SARS-CoV-2, various systemic and local symptoms, so-called post-COVID-19 syndrome (PCS) (Long Haul Syndrome), may even appear up to 9 months after getting well. Symptoms can change or come back and cannot be explained in an alternative way. In total, 80% of patients report fatigue, 59% neurological symptoms, 34% psychological problems, 59% respiratory problems, sore throat, cough, runny nose, diarrhea, nausea, sweating, skin rashes, 34% difficulties in activities of daily living (ADLs), palpitations, joint and muscle pain, menstrual disorders, and loss of taste and smell. Severe COVID-19 and hospitalization may prompt the emergence of postintensive care syndrome or posttraumatic stress disorder. 8 9 10 Many oral symptoms have also been reported, such as dryness, follicular lesions, 11 12 13 recurrent ulcerations and erosions, 14 mucositis, 15 exanthema, 16 cheilitis, 17 mycosis 18 aphthous-like changes, 19 pustules, cracked or papillae-free tongue, spots, papules, change in pigmentation, halitosis, leukoplakia, hemorrhagic crusts, ecchymosis, edema, erythema, and spontaneous bleeding. 20 21 22 23

According to the available data, oral symptoms appeared in 68% of patients, including 49% of women and 51% of men. Patients in old age and/or with greater severity of SARS-CoV-2 had more extensive lesions in the oral cavity. 6

PCS observed in the oral cavity may be the result of stress, inadequate oral hygiene, vasculitis, multiorgan disorders, opportunistic infections, reinfection, or dysfunction of the immune system. 15 24

The aim of the study is to present six selected cases of the most common symptoms of long COVID in the oral cavity of patients with a history of SARS-CoV-2 infection.

## Case Reports

Until mid-2021 the clinic “Stomatologia Rafałowicz” provided 1,256 consultations of patients who had been infected with SARS-CoV-2 in the period from 2 to 6 months before the visit.

Among the studied patients, 32% of them had discoloration, ulceration, and hemorrhagic changes on the oral mucosa, 29.69% had mycosis located on the tongue, 25.79% of patients had unilateral (more often left-sided) aphthous-like lesions on the hard palate, and in 12.5% atrophic cheilitis was observed. During the anamnesis, approximately 60% of patients reported salivary secretory disorders in the initial period of infection, which in 6.68% prolonged up to 4 months after systemic symptoms disappeared.

Approximately 36% of patients did not agree to the proposed treatment. As a result, they only received recommendations on the use of oral hygiene products and received weekly check-ups. In this group of patients, most pathological changes spontaneously cleared after 3 weeks. The changes located on the palate took the longest to heal.

About 30% of patients over 70 years of age with comorbidities or hospitalized had more extensive and severe changes in the oral cavity that persisted for a long time after infection.

In an extreme case, an aphthous-like lesion persisted for 6 months.

The case reports were guided by using the CARE checklist. 25

On all six patients' panoramic radiographs were performed which did not show pathologic changes in the bone tissue.

### Case 1

On June 21, 2021, 43-year-old man, businessman, came for a routine visit to the dental office. The patient was infected with SARS-CoV-2 in December 2020 and there are no comorbidities. The patient was in quarantine for 3 weeks and had the following symptoms: fever, malaise, taste disorders, anosmia, and pneumonia. In the acute phase he was taking steroids. He reported PCS symptoms: fatigue, sleep disturbances, respiratory problems, and sweating. He is under the control of a pulmonologist.

The patient has a balanced diet, is not overweight, and does not smoke or drink alcohol.

**Fig. 1:**
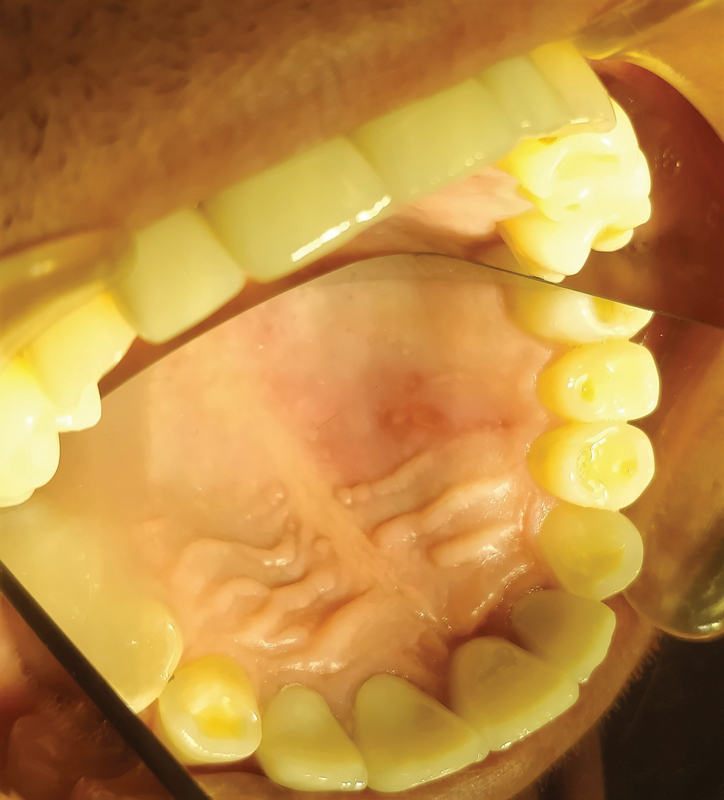
Aphthous-like lesion with red inflamed periphery.

During an intraoral examination, unilateral aphthous-like lesions with inflamed limbus were found on the left side of the hard palate ( Fig. 1 ). Status of oral hygiene was without problems. The patient did not report any pain during that time, but the only discomfort was due to the existing lesion.

A semiconductor laser therapy with the Smart bio stimulation function was used (Lasotronix)—five treatments at 3-day intervals and it was recommended to use a mouthwash containing chlorhexidine three times a day for a period of 14 days. The aphthous-like lesions persisted for 192 days after the end of the acute phase of infection. The patient is under periodic dentist observation. Local lesions have healed, but general PCS symptoms persist. The patient is diagnosed with pulmonary calcification.

### Case 2

On June 9, 2021, a 72-year-old man, pensioner, came for a dental consultation due to persistent bleeding in the oral cavity, difficulty swallowing, and burning lips. The patient was infected with SARS-CoV-2 in April 2021 and has hypertension and insulin resistance. Due to the onset of dyspnea, persistent diarrhea, and vomiting, the patient was hospitalized. Fifty-nine days after COVID-19 subsided, he reported PCS symptoms: chronic fatigue, problems with concentration, palpitations, shortness of breath, and drenching sweats.

The patient is on a diabetic diet, is not overweight, does not smoke or drink alcohol, and shows normal ADLs. He is taking drugs for hypertension, controlling heart rate, and diabetes.

**Fig. 2:**
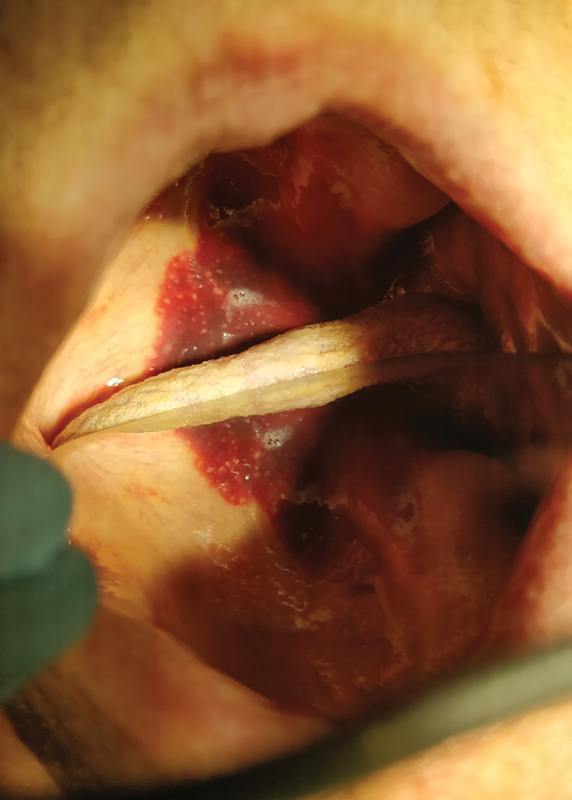
Hemorrhagic changes, extensive wounds, and spontaneous bleeding.

Intraoral examination revealed hemorrhagic changes on the palate, extensive wounds, spontaneous bleeding, and cheilitis ( Fig. 2 ). The patient was directed for specialist treatment to the Department of Periodontology and Oral Diseases. The prognosis is difficult to determine.

### Case 3

On March 11, 2021, a 59-year-old woman, accountant, came to the dental office due to persistent dry mouth. The patient was infected with SARS-CoV-2 in January 2021 and has rheumatoid arthritis. Due to dyspnea, she was hospitalized for 7 days including connection to a breathing aid device. Fifty-six days after the acute phase of COVID-19, she reported PCS symptoms: fatigue, irritability, trouble sleeping and concentrating, and sweating.

The patient eats a diet rich in fats and carbohydrates, is obese, does not smoke or drink alcohol, and shows normal ADLs.

**Fig. 3:**
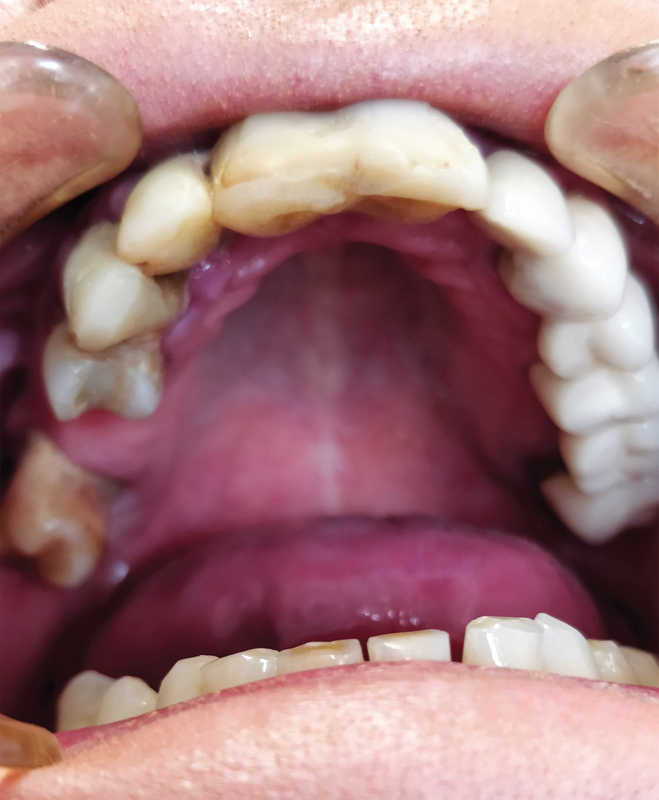
Intense purple-red color throughout the whole mouth.

During an intraoral examination, the whole mucosa was intensely red-purple, and the surface of the tongue was smooth ( Fig. 3 ). To reduce dryness, the patient was recommended to use saliva-stimulating tablets SST (Sinclair Pharmaceuticals), Kserostemin (artificial saliva) (Aflofarm), and mouthwash with chlorhexidine three times a day for a period of 14 days. After 2 weeks, the secretion of saliva improved, the feeling of dry mouth and the intensity of the oral mucosa discoloration decreased ( Fig. 3 ). The dental prognosis is good, whereas the overall prognosis is difficult to determine.

### Case 4

On June 7, 2021, a 48-year-old man, businessman, came to the dental office due to the loss of the retention of prosthetic crowns on teeth 11 and 12. The patient was infected with SARS-CoV-2 in January 2021with a loss of smell and taste and fever for 9 days and there are no comorbidities.

The patient was in quarantine. A total of 129 days after the acute phase of COVID-19, he reported PCS symptoms: concentration difficulty, amnesia, shortness of breath, palpitations, and sweating.

The patient has a balanced diet, is not overweight, and does not smoke. He leads an active lifestyle and drinks alcohol with restraint.

**Fig. 4:**
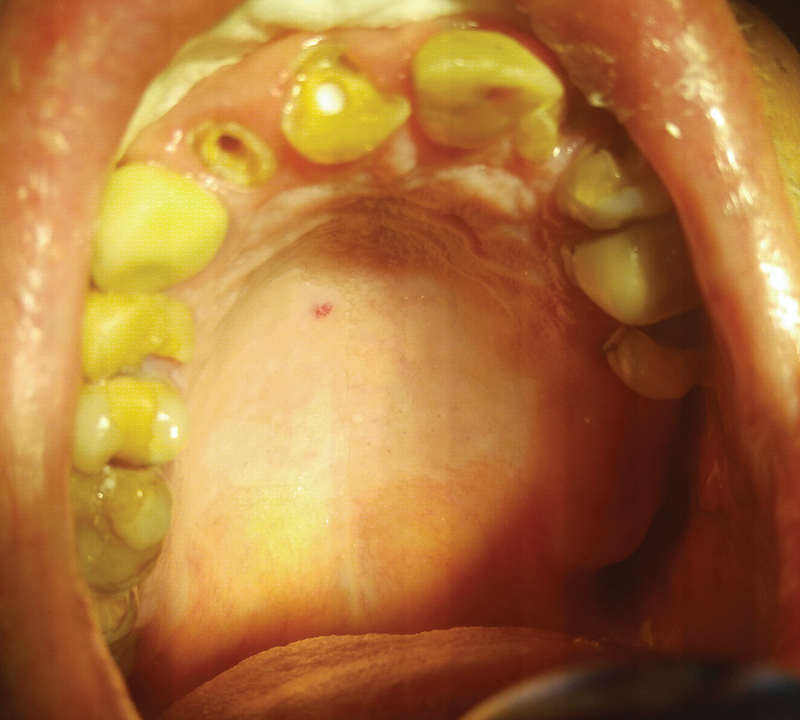
Change of the angioma type.

During the intraoral examination, a lesion of the angioma type was found on the right side of the palate. According to the patient's observation, the change decreased spontaneously after 3 months ( Fig. 4 ). As no further improvement was observed in the next 2 months, the patient was directed for specialist treatment to the Department of Periodontology and Oral Diseases.

The prognosis for dental treatment is good and the change has significantly decreased. Overall prognosis is difficult to determine.

### Case 5

On May 25, 2021, a 66-year-old woman, pensioner, came to the dental office due to a broken tooth 12. The patient was infected with SARS-CoV-2 in January 2021 in a mild form with loss of smell and taste for 6 days and there are no comorbidities. A total of 116 days after COVID-19, she reported PCS symptoms: chronic fatigue, depression, palpitations, and sweating.

The patient has a balanced diet, is not overweight, does not smoke or drink alcohol, and shows medium ADLs.

**Fig. 5:**
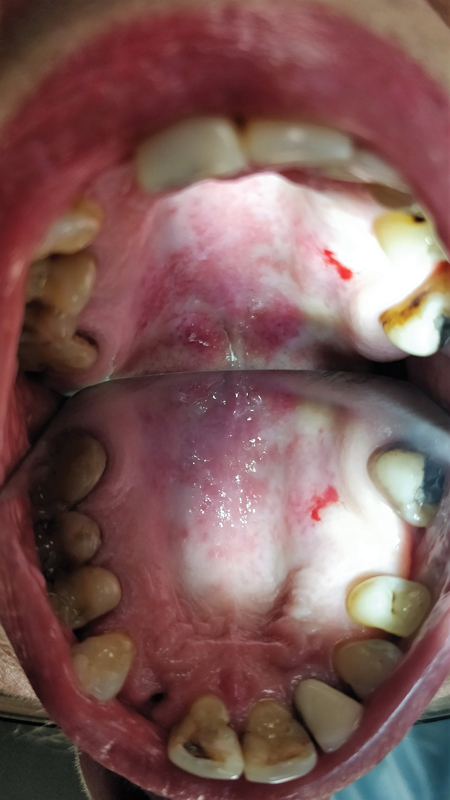
Hemorrhagic changes on the palate with spontaneous bleeding.

Intraoral examination revealed extensive changes of the vascular type on the hard palate with spontaneous bleeding without pain ( Fig. 5 ). The patient was directed for specialist treatment to the Department of Periodontology and Oral Diseases. The local changes disappeared after 60 days. Overall prognosis is difficult to determine.

### Case 6

**Fig. 6:**
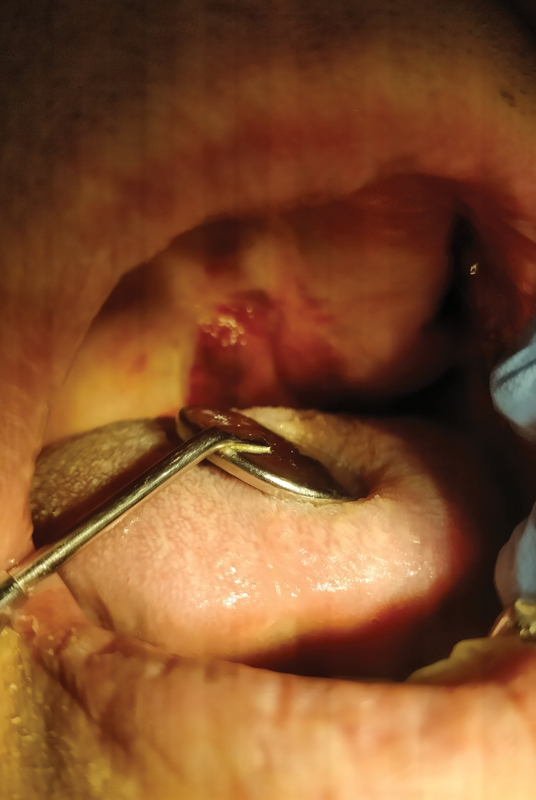
Vascular ecchymosis, cheilitis, and mycosis of the tongue.

On June 3, 2021, a 71-year-old man, pensioner, came to the dental office due to persistent bleeding in the mouth and dry lips ( Fig. 6 ). The patient was infected with SARS-CoV-2 in April 2021 in a mild form with loss of smell and taste for 7 days and has hypertension, type 1 diabetes, and allergies. Due to the onset of dyspnea, persistent diarrhea, and vomiting, he was hospitalized. A total of 46 days after COVID-19 subsided, he reported PCS symptoms: disturbance in concentration and taste, shortness of breath, and vomiting.

The patient is on a diabetic diet, is not overweight, does not smoke or drink alcohol, and shows limited ADLs. He is taking drugs for hypertension, insulin injections, and antiallergic agents.

Intraoral examination revealed mycosis of the tongue, extensive lesions on the palate, spontaneous bleeding, and cheilitis. For 10 days, the antifungal Nystatin (Teva) antibiotic was administered at a dose of 100,000 IU/mL four times a day along with vitamin A + E + F (Gorvita) ointment on the lips. Due to persistent spontaneous bleeding and hemorrhagic changes on the palate, the patient was directed for specialist treatment to the Department of Periodontology and Oral Diseases. Local changes and general symptoms of PCS persist.

## Discussion

Clinical studies conducted in a group of patients after SARS-CoV-2 infection, as well as data from the literature, indicate the complexity of the long COVID problem. It was observed that each case is characterized by an individual course and different general and local symptoms. Various clinical symptoms in the oral cavity not only occur during infection but may also persist for many months after systemic symptoms have subsided. Aphthous-like lesions and small ulcers that underwent laser therapy healed after four to five treatments. The mycotic lesions resolved after 10 days of using the Nystatin antibiotic. In patients with impaired salivary secretion, drugs for stimulation were used for 14 days, which allowed for improvement of secretion.

Based on many observations, it was found that the symptoms of long COVID in the oral cavity are associated with a decrease in the body's immunity, stress, and the general health of the patient. 5 6 22 24

It is important that patients who report a history of SARS-CoV-2 infection undergo a detailed intraoral examination to detect any symptoms that may be related to it. It has been observed that pathological changes in the oral cavity may persist for 2 to 6 months after the infection. According to Melo Neto et al, changes in the oral cavity may appear 10 to 42 days after the onset of systemic symptoms and disappear spontaneously or within 3 weeks after treatment. 5 According to Paradowska, aphthous-like changes disappear after 5 to 15 days and resembling erythema multiforme changes after 2 to 4 weeks. 6 Other authors found that aphthous stomatitis usually resolved after 9 days of using only oral hygiene products. 22 The observed, spontaneous resolution of oral disease symptoms in 36% of patients confirms the observations of other authors. 5 6 However, patients who do not consent to treatment should be kept under control because of the possibility of opportunistic infections.

Localization of lesions on the palate, tongue, and salivary glands is associated with a high expression of ACE2 receptors, therefore, the oral cavity is a potential high-risk area of SARS-CoV-2 infection. According to Sabino-Silva et al, the salivary glands are the reservoir of the virus, and saliva plays a key role in the human-to-human transmission of the virus. 3

According to Dziedzic and Wojtyczka, it is not possible to clearly establish a causal relationship between the past infection and the occurrence of changes in the oral cavity. 21 As with human immunodeficiency virus infection, patients with SARS-CoV-2 are more likely to develop oral lesions associated with immunosuppression. Lack of oral hygiene in hospitalized patients in connection to a breathing aid device is a likely cause of opportunistic infections, e.g., mycosis. Recurrent herpes simplex virus infections, nonspecific mouth ulcers, drug eruptions, dysgeusia, xerostomia, and gingivitis and ulceration occur because of the impaired immune system or oral mucosa compliance. 21 23

The coexistence of other underlying diseases not related to past SARS-CoV-2 infection cannot be ruled out, as mentioned by other authors. Oral mucosa lesions in people with COVID-19 can mimic other oral diseases, such as reactive, vascular, and immune disorders, which are necessary for their differentiation for correct diagnosis and clinical management in patients with SARS-CoV-2. 7 15 21 22 23 24

The observed clinical picture and the results of histological examinations suggest the possibility of primary or secondary changes in the oral cavity related to vascular and hematological damage. Recently published studies on changes in the oral mucosa during SARS-CoV-2 confirm the association with organic damage or complications of thrombocytopenia, antithrombotic treatment, disseminated intravascular coagulation, and systemic inflammation. 11 According to Martín Carreras-Presas et al, the presence of long COVID symptoms results from primary or secondary vascular and hematological changes and lymphocytic thrombotic arteritis. 12

In total, 25% of patients with hemorrhagic lesions and coexisting mycosis admitted to the clinic were directed for specialist treatment to the Department of Periodontology and Oral Diseases.

According to some authors, it has not been established whether SARS-CoV-2 infection is the direct cause or a factor predisposing to the occurrence of lesions in the oral cavity. 24 Martín Carreras-Presas et al believe that systemic deterioration of health, acute onset, and multidrug therapy may be the cause of pathological changes in the oral cavity. 12 24 Amorim Dos Santos et al associate the occurrence of secondary mouth ulcers with a similar immune response as in the case of other viral infections. 13 SARS-CoV-2 may therefore overactivate the inflammatory immune response, leading to a cytokine storm and immune exhaustion, which may result in early oral lesions. 22 23 24

It is also possible that the observed cases may indicate reinfection, which may occur many months after the primary infection. 16 22 23

## Conclusion

In patients after the SARS-CoV-2 infection and suspected of this infection, a detailed intraoral examination should be performed, and the patient must be obligatorily monitored for a minimum period of 6 months. Depending on the patient's clinical condition, changes in the oral cavity require observation, basic or specialist treatment. In the case of changes in the cavity without pain symptoms, observation should be made for approximately 4 weeks and wait for the spontaneous regression of the changes. However, when pain occurs, a good solution is to use laser biostimulation. In the case of complex pathological changes occurring in the oral cavity, the patient should be directed for specialist treatment.
